# Evaluation of Metabolic Defects in Fatty Acid Oxidation Using Peripheral Blood Mononuclear Cells Loaded with Deuterium-Labeled Fatty Acids

**DOI:** 10.1155/2019/2984747

**Published:** 2019-02-07

**Authors:** Miori Yuasa, Ikue Hata, Keiichi Sugihara, Yuko Isozaki, Yusei Ohshima, Keiichi Hara, Go Tajima, Yosuke Shigematsu

**Affiliations:** ^1^Department of Pediatrics, Faculty of Medical Sciences, University of Fukui, 23-3 Matsuoka-Shimoaizuki, Eiheijai-cho, Yoshida-gun, Fukui 910-1193, Japan; ^2^Department of Pediatrics, National Hospital Organization Kure Medical Center, 3-1 Aoyama-cho, Kure-shi, Hiroshima 737-0023, Japan; ^3^Division of Neonatal Screening, Research Institute, National Center for Child Health and Development, 2-10-1 Okura, Setagaya-ku, Tokyo 157-8535, Japan

## Abstract

Because tandem mass spectrometry- (MS/MS-) based newborn screening identifies many suspicious cases of fatty acid oxidation and carnitine cycle disorders, a simple, noninvasive test is required to confirm the diagnosis. We have developed a novel method to evaluate the metabolic defects in peripheral blood mononuclear cells loaded with deuterium-labeled fatty acids directly using the ratios of acylcarnitines determined by flow injection MS/MS. We have identified diagnostic indices for the disorders as follows: decreased ratios of d_27_-C14-acylcarnitine/d_31_-C16-acylcarnitine and d_23_-C12-acylcarnitine/d_31_-C16-acylcarnitine for carnitine palmitoyltransferase-II (CPT-II) deficiency, decreased ratios of d_23_-C12-acylcarnitine/d_27_-C14-acylcarnitine for very long-chain acyl-CoA dehydrogenase (VLCAD) deficiency, and increased ratios of d_29_-C16-OH-acylcarnitine/d_31_-C16-acylcarnitine for trifunctional protein (TFP) deficiency, together with increased ratios of d_7_-C4-acylcarnitine/d_31_-C16-acylcarnitine for carnitine palmitoyltransferase-I deficiency. The decreased ratios of d_1_-acetylcarnitine/d_31_-C16-acylcarnitine could be indicative of *β*-oxidation ability in patients with CPT-II, VLCAD, and TFP deficiencies. Overall, our data showed that the present method was valuable for establishing a rapid diagnosis of fatty acid oxidation disorders and carnitine cycle disorders and for complementing gene analysis because our diagnostic indices may overcome the weaknesses of conventional enzyme activity measurements using fibroblasts or mononuclear cells with assumedly uncertain viability.

## 1. Introduction

Fatty acid oxidation disorders and carnitine cycle disorders are characterized by infant sudden death or acute encephalopathy due to hypoglycemia and hyperammonemia during fasting events and are designated as important target disorders in newborn screening (NBS) by tandem mass spectrometry (MS/MS), in which acylcarnitines in dried blood spots (DBS) are measured. However, the severity of these disorders varies markedly, and screening markers in DBS may show slight abnormalities in some mild cases. In Japan, there are reports of some cases with episodes of severe metabolic crises having been missed in NBS using concentrations of C16 and C18:1 acylcarnitines as screening markers for carnitine palmitoyltransferase-II (CPT-II) deficiency; therefore, the ratios of (C16+C18:1)/C2 and C14/C3 have recently been introduced as new screening markers [1]. Moreover, it may be necessary to lower the cutoff values for these markers in order to avoid false negatives resulting in higher false-positive cases. Accordingly, time-consuming and costly confirmation tests, such as enzyme activity measurement using cultured skin fibroblasts or gene analysis, may be needed.

Serum acylcarnitines are first measured in positive cases of NBS and high-risk cases of fatty acid oxidation disorders and carnitine cycle disorders. However, acylcarnitine profiles in serum do not always provide diagnostic results for evaluating these disorders [2, 3]. Thus, the additional diagnostic measures, including probe assays using cultured skin fibroblasts [4, 5] and tracer analysis using whole blood loaded with deuterium-labeled palmitate [6], have been developed. In the former assay, invasive procedures are required to obtain skin samples, several days of cell culture are needed, and acylcarnitine changes in the culture medium, not in cultured cells, are evaluated. The latter is a simple measurement of the concentrations of deuterium-labeled acylcarnitines in whole blood after incubation but does not seem to yield satisfactory data for evaluating abnormalities in some disorders.

Therefore, we developed a rapid, noninvasive, confirmative diagnostic test using MS/MS, which has been used in NBS. Peripheral blood mononuclear cells (PBMCs) were incubated for two hours in a buffer containing deuterium-labeled palmitic acid, which was incorporated into the collected cells. Acylcarnitines in the incubated cells were analyzed using flow injection MS/MS to observe the metabolic process of labeled palmitic acid in the cells directly. In this experiment, the accumulation of the deuterium-labeled acylcarnitines is thought to reflect abnormalities in metabolic processes. Because palmitic acid with deuterium instead of all hydrogens was used, labeled acetylcarnitine from labeled acetyl-CoA, the product of *β*-oxidation, is expected to function as a marker in the evaluation of *β*-oxidation ability in incubated cells. Then, we evaluated the ratios of the labeled acylcarnitines, rather than the concentrations, in order to precisely identify the process abnormalities. In PBMCs, the activities of enzymes related to these disorders vary widely, even in healthy controls [1, 7]. Enzyme activities in PBMCs are typically corrected according to the number of the cells used in the assay, not according to the assumedly variable viability of the cells. Thus, the difficulties encountered in enzyme assays using PBMCs due to changes in cell viability could be overcome by the ratios proposed here as diagnostic indices.

Carnitine palmitoyltransferase-I (CPT-I) deficiency results from abnormalities in the CPT-I-A protein in the liver [8]. Although CPT-I-A is expressed in lymphocytes as well as in the liver, the CPT-I activities have been typically measured using cultured skin fibroblasts [9, 10], rather than PBMCs. Enzyme assays using lymphocytes to test for CPT-I deficiency and trifunctional protein (TFP) deficiency/long-chain hydroxyacyl-CoA dehydrogenase (LCAHD) deficiency have been reported from the limited laboratories [11], despite our recent development of enzyme assays for CPT-II, very long-chain acyl-CoA dehydrogenase (VLCAD), and medium long-chain acyl-CoA dehydrogenase (MCAD) using lymphocytes [1, 7, 12]. Our test could make the diagnosis of CPT-I deficiency and TFP deficiency/LCAHD deficiency, together with the diagnosis of CPT-II deficiency and VLCAD deficiency, both simpler and less invasive.

Accordingly, in this study, we used this method to test patients with carnitine cycle disorders and long-chain fatty acid *β*-oxidation disorders and examined the significance and usefulness of this method as a diagnostic tool.

## 2. Materials and Methods

### 2.1. Materials

#### 2.1.1. Biological Samples

Blood samples were collected from 3 patients with CPT-I deficiency, 6 patients with CPT-II deficiency, 5 patients with VLCAD deficiency, 3 patients with TFP deficiency, and 31 healthy adult controls. The data from 8 controls were used for examination of CPT-I deficiency, and the remaining data were used for examining the other 3 disorders.  Table 1 lists the clinical forms of the disorders, the values of both the screening markers of acylcarnitine in DBS for NBS and the diagnostic markers of acylcarnitine in serum at onset, and enzyme activities. All patients, except those with CPT-I deficiency, had been tentatively diagnosed by serum acylcarnitine analysis with abnormalities specific to the disorder, and the diagnosis was confirmed by gene analysis, including CPT-I deficiency. Enzyme activities were found to be decreased in most of the patients with VLCAD deficiency. The ages of the patients ranged from 0 to 32 years (median: 5 years) when the blood samples were collected. Both patient no. 1 and patient no. 3 with CPT-II deficiency were missed in the NBS based on the former cutoff value. Patient no. 6 with CPT-II deficiency had a mild elevation in C16-acylcarnitine concentration at onset, as well as gene mutations found in patients with the mild myopathic form of the disease [13].

#### 2.1.2. Chemicals

The stable isotope-labeled acylcarnitines used in this study were synthesized by our laboratory [15], except for [^2^H_3_] (d_3_)-pentadecanoylcarnitine and d_3_-hexadecanoylcarnitine, which were purchased from VU Medical Center Metabolic Laboratory (Amsterdam, The Netherlands). [^2^H_31_] d_31_-palmitic acid and [^2^H_15_] d_15_-octanoic acid were from Cambridge Isotope Laboratories Inc. (Tewksbury, MA, USA). L-Carnitine was from Wako Pure Chemical Industries (Osaka, Japan), and fatty acid-free bovine serum albumin (BSA) was from British BioCell International Ltd. (Newport, UK). Phosphate-buffered saline (PBS) and Dulbecco's PBS (DPBS) were purchased from Nissui Pharmaceutical Co. Ltd. (Tokyo, Japan) and Nacalai Tesque (Kyoto, Japan), respectively, and Ficoll-Paque solution was from GE Healthcare (Tokyo, Japan).

### 2.2. Methods

#### 2.2.1. Sample Preparation

A mixture of 5 mL heparinized whole blood and 5 mL of D-PBS was layered on Ficoll-Paque solution and centrifuged. The collected PBMC layer was washed with PBS and then suspended in 0.5 mL D-PBS. L-Carnitine and d_31_-hexadecanoic acid (0.5 mg/mL) in 3% fatty acid-free BSA were added to the cell suspension and then incubated at 37°C for 2 h. In order to evaluate CPT-I deficiency, d_15_-octanoic acid (0.5 mg/mL) in 3% fatty acid-free BSA was also added to evaluate cell availability. The incubation mixture was centrifuged, and PBMCs were washed with PBS. The washed PBMCs were suspended in methanol and homogenized with a glass homogenizer. The supernatant collected after centrifugation was spiked with methanol solution of stable isotope-labeled acylcarnitines as internal standards.

#### 2.2.2. Acylcarnitine Analysis by MS/MS

Flow injection and electrospray ionization MS/MS (ESI-MS/MS) analysis was performed using a model API 4000 LC/MS/MS system (AB Sciex, Tokyo, Japan). We used a model LC-10AVP HPLC system and a model SIL-20AC autoinjector (Shimadzu, Kyoto, Japan). Five microliters of sample was introduced into the liquid chromatography (LC) flow of acetonitrile/water (4 : 1) with 0.05% formic acid. Positive ion MS/MS analysis was performed in precursor ion scan mode with an 85 m/z of product ion. The data were recorded for 0.7 min after every sample injection, and the recorded intensities of the designated ions were averaged for quantification using ChemoView Software (AB Sciex). The concentrations of hydroxyhexadecanoylcarnitine (C16-OH) and hydroxytetradecanoylcarnitine (C14-OH) were calculated using the ion intensities of d_3_-hexadecanoylcarnitine and d_3_-tetradecanoylcarnitine, respectively.

#### 2.2.3. Diagnostic Indices of Each Disorder

Based on the metabolic pathway of d_31_-C16-acylcarnitine (d_31_-hexadecanoylcarnitine, d_31_-C16) and involvement of each enzyme, we calculated the following ratios reflecting causative enzyme activities: the ratios of d_27_-tetradecanoylcarnitine (d_27_-C14)/d_31_-C16, d_23_-dodecanoylcarnitine (d_23_-C12)/d_27_-C14, and d_23_-C12/d_31_-C16 for CPT-II deficiency and VLCAD deficiency and that of d_29_-hydroxyhexadecanoylcarnitine (d_29_-C16-OH)/d_31_-C16 for TFP deficiency.

CPT-I-deficient cells can barely produce d_31_-C16 from d_31_-hexadecanoic acid in mitochondria. However, CPT-I-deficient cells can incorporate d_15_-octanoic acid and thereby produce labeled short- to medium-chain acylcarnitines. Because cells with decreased viability may show poor production of d_31_-C16-acylcarnitine, d_15_-octanoic acid was added together with d_31_-hexadecanoic acid to the cell culture to discriminate CPT-I deficiency from decreased cell viability. Thus, the ratios of d_7_-butyrylcarnitine (d_7_-C4)/d_31_-C16 in CPT-I-deficient cells and control cells loaded with d_31_-hexadecanoic acid and the ratios in those loaded with both d_15_-octanoic acid and d_31_-hexadecanoic acid were tested for CPT-I deficiency.

In addition, the amount of d_1_-acetylcarnitine (d_1_-C2), a product from d_31_-palmitic acid through *β*-oxidation, was measured to investigate the correlation with *β*-oxidation ability.

#### 2.2.4. Statistical Analysis

Data in controls are expressed as medians and interquartile ranges (25th–75th percentile). The differences in each ratio between patients and controls were analyzed using the Mann-Whitney tests, and the correlations between ratios in patients were analyzed using the Spearman's rank correlation coefficient. A *p* value of less than 0.05 was considered to denote statistical significance. All analyses were performed using EZR (Saitama Medical Center, Jichi Medical University, Saitama, Japan), a graphical user interface for R (the R Foundation for Statistical Computing, Vienna, Austria) [16].

## 3. Results

**Figure 1:**
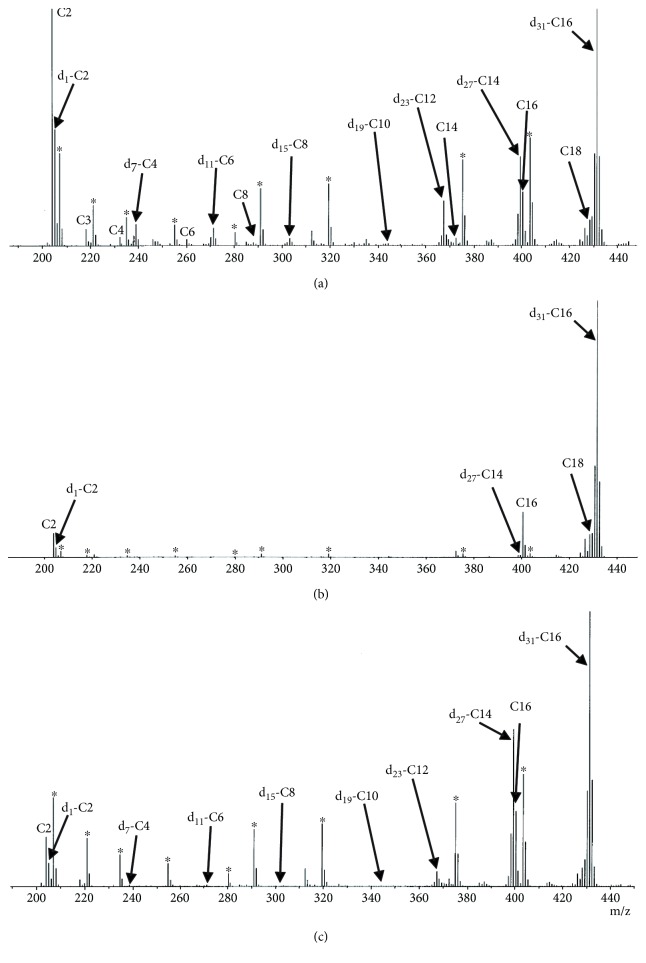
Precursor ion mass spectra observed in acylcarnitine analysis by MS/MS. Homogenized PBMCs loaded with d_31_-hexadecanoic acid were subjected to acylcarnitine analysis. (a) Healthy control, (b) CPT-II deficiency, and (c) VLCAD deficiency. ^∗^Stable isotope-labeled acylcarnitines as internal standards.

Data from the acylcarnitine analysis of the fatty acid oxidation test for each patient are shown in Tables S1 and S2 in Supplementary Materials. A series of labeled acylcarnitines, from d_31_-C16 to d_1_-C2, was detected in PBMCs loaded with d_31_-hexadecanoic acid in healthy controls (Figure 1(a)). PBMCs from patients with CPT-II deficiency showed apparent decreases in metabolic products from d_27_-C14, and that from patients with VLCAD deficiency showed decreases in metabolic products from d_23_-C12 (Figures 1(b) and 1(c)). We analyzed defects in the metabolic pathway in patient samples using the ratios of metabolic products.

**Figure 2:**
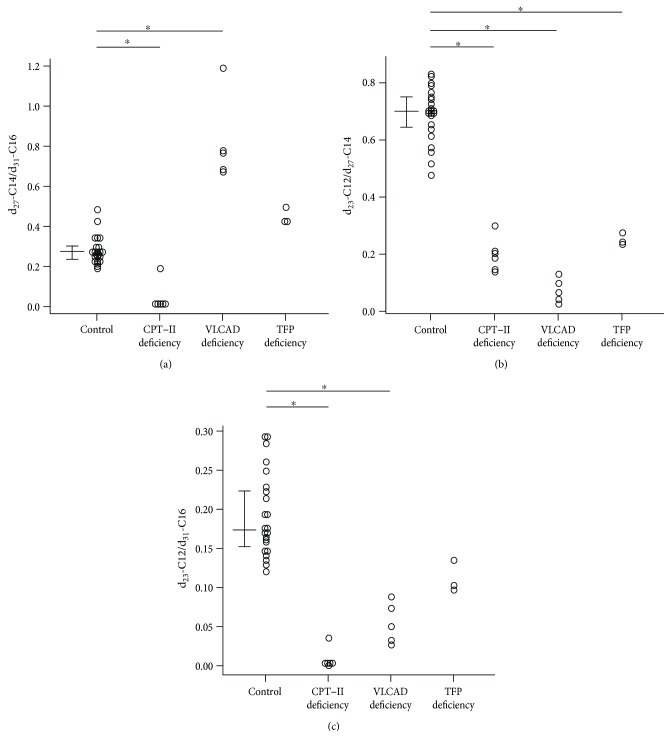
Comparison of diagnostic ratios between healthy controls and patients with CPT-II deficiency, VLCAD deficiency, and TFP deficiency. (a) d_27_-C14/d_31_C16, (b) d_23_-C12/d_27_-C14, and (c) d_23_-C12/d_31_-C16. ^∗^ *p* < 0.001, significant difference between the control and each disorder. The horizontal lines in controls represent the medians and interquartile ranges (25th–75th percentile).

As shown in Figure 2(a), the ratio of d_27_-C14/d_31_-C16 was significantly lower in patients with CPT-II deficiency and higher in patients with VLCAD deficiency compared with healthy controls. The ratios of d_23_-C12/d_27_-C14 for all examined patients were lower than those in controls (Figure 2(b)). The ratios of d_23_-C12/d_31_-C16 in patients with CPT-II deficiency and VLCAD deficiency but not in patients with TFP deficiency were lower than those in controls (Figure 2(c)). Thus, CPT-II deficiency was characterized by decreased d_27_-C14/d_31_-C16, d_23_-C12/d_27_-C14, and d_23_-C12/d_31_-C16 ratios, whereas VLCAD deficiency was characterized by increased d_27_-C14/d_31_-C16 and decreased d_23_-C12/d_27_-C14 and d_23_-C12/d_31_-C16 ratios.

**Figure 3:**
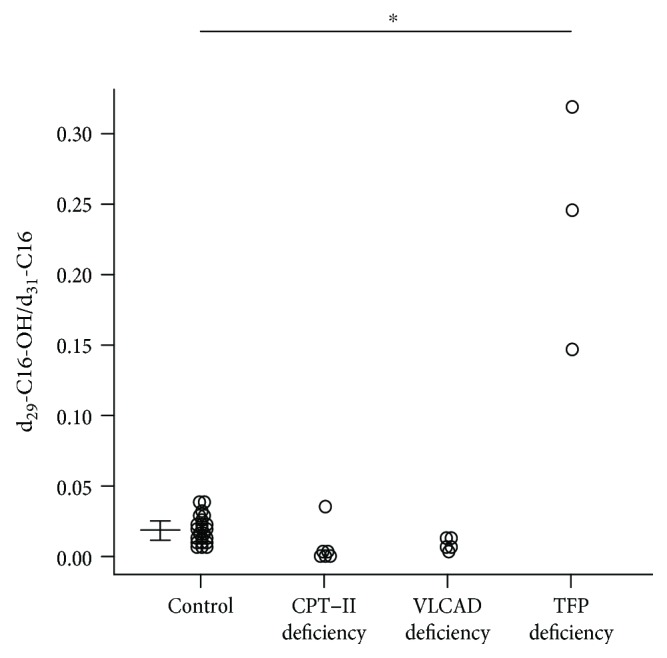
Comparison of diagnostic ratios of d_29_-C16-OH/d_31_-C16 between healthy controls and patients with CPT-II deficiency, VLCAD deficiency, and TFP deficiency. ^∗^ *p* < 0.001, significant difference between control and TFP deficiency. The horizontal lines in controls represent the medians and interquartile ranges (25th–75th percentile).

Analysis of TFP deficiency showed significantly elevated ratios of d_29_-C16-OH/d_31_-C16 (Figure 3). There were no significant differences in this ratio among CPT-II deficiency, VLCAD deficiency, and controls.

**Figure 4:**
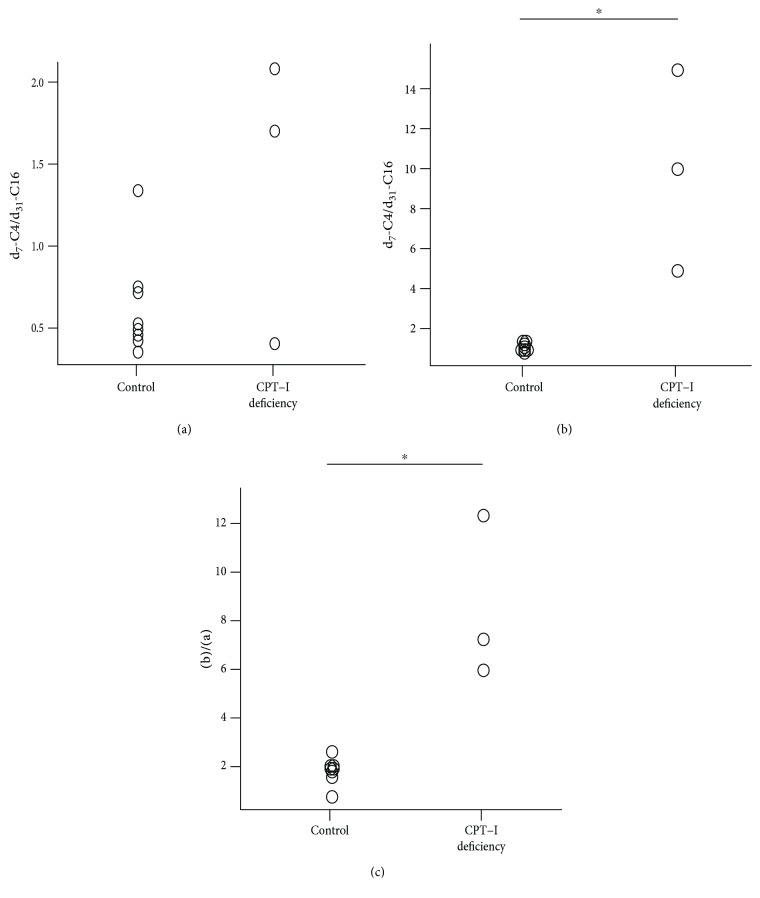
Comparison of diagnostic ratios and indexes between controls and patients with CPT-I deficiency. (a) d_7_-C4/d_31_-C16 after loading of d_31_-hexadecanoic acid alone (*p* = 0.38). (b) d_7_-C4/d_31_-C16 after loading of d_15_-octanoic acid and d_31_-hexadecanoic acid (^∗^ *p* = 0.012). (c) The adjusted ratio of d_7_-C4/d_31_-C16, corresponding to (b)/(a) (^∗^ *p* = 0.012).

After loading of d_31_-hexadecanoic acid alone, PBMCs from patients with CPT-I deficiency produced much less d_31_-C16 than those from controls. There were no significant differences in the ratios of d_7_-C4/d_31_-C16 between controls and patients with CPT-I deficiency (Figure 4(a)). After loading of a mixture of d_31_-hexadecanoic acid and d_15_-octanoic acid, however, PBMCs from patients with CPT-I deficiency metabolized d_15_-octanoic acid but not d_31_-hexadecanoic acid, resulting in increased d_7_-C4 and unchanged d_31_-C16 levels. Thus, the ratio of d_7_-C4/d_31_-C16 was significantly higher in patients with CPT-I deficiency than in controls (Figure 4(b)). We adjusted the ratio after loading of the mixture by dividing by the ratio after loading of d_31_-hexadecanoic acid alone. As shown in Figure 4(c), the adjusted ratio of d_7_-C4/d_31_-C16 was significantly higher in patients with CPT-I deficiency than in controls.

**Figure 5:**
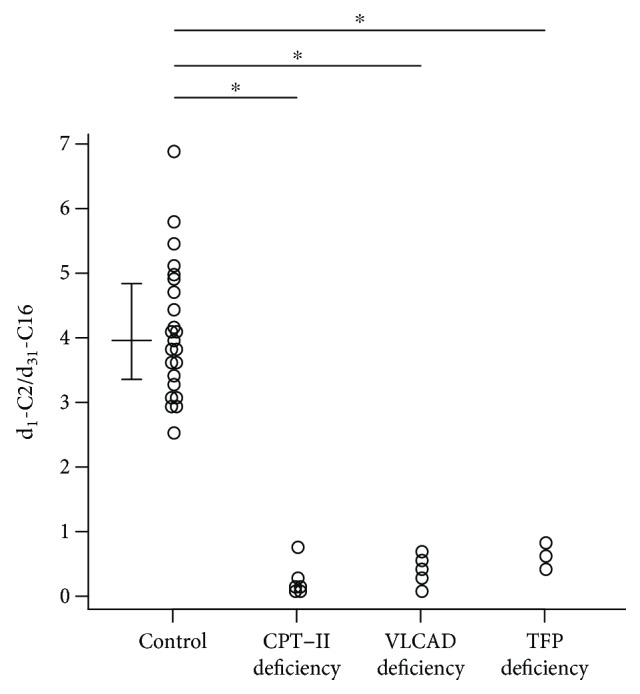
Comparison of the *β*-oxidation ability index (the ratios of d_1_-C2/d_31_-C16) between healthy controls and CPT-II deficiency, VLCAD deficiency, and TFP deficiency. ^∗^ *p* < 0.001, significant difference between the control and each disorder. The horizontal lines in controls represent the medians and interquartile ranges (25th–75th percentile).

**Figure 6:**
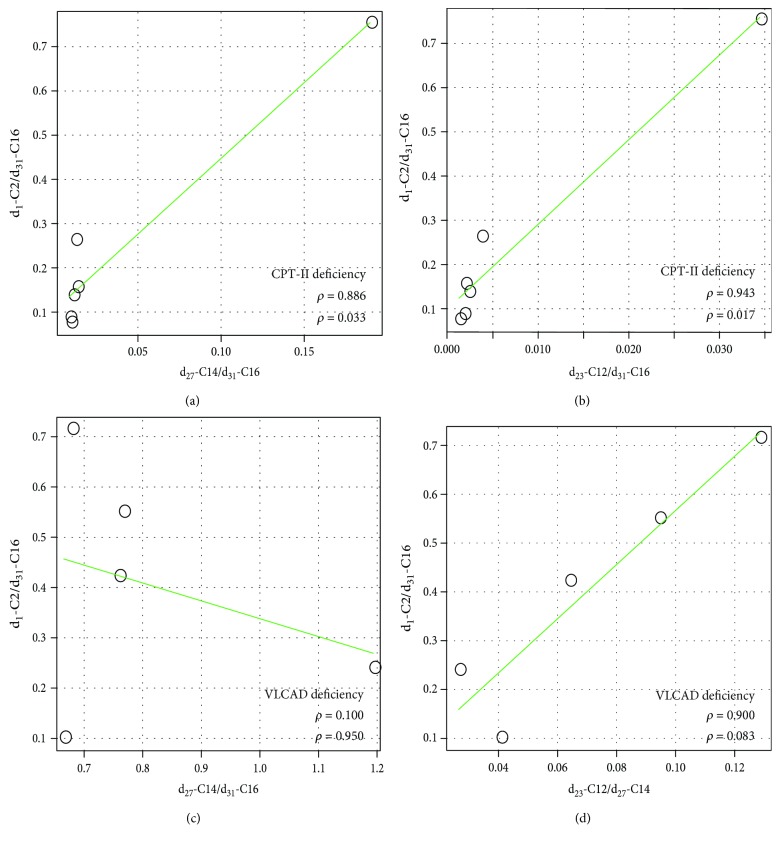
Correlation between the ratios of d_1_-C2/d_31_-C16 and the diagnostic ratios. Correlations between d_1_-C_2_/d_31_-C16 and d_27_-C14/d_31_-C16 (a) and between d_1_-C_2_/d_31_-C16 and d_23_-C12/d_31_-C16 (b) in CPT-II deficiency. Correlations between d_1_-C2/d_31_-C16 and d_27_-C14/d_31_-C16 (c) and between d_1_-C2/d_31_-C16 and d_23_-C12/d_27_-C14 (d) in VLCAD deficiency. The Spearman's rank correlation coefficient *ρ* and *p* values are indicated.

Because d_1_-C2 is produced during each cycle of *β*-oxidation, the total amounts of d_1_-C2 could reflect *β*-oxidation ability. The ratios of d_1_-C2/d_31_-C16 were significantly decreased in patients with CPT-II deficiency, VLCAD deficiency, and TFP deficiency compared with controls, indicating impairment of the *β*-oxidation pathway (Figure 5). As shown in Figure 6, CPT-II deficiency had positive correlations between d_1_-C2/d_31_-C16 and d_27_-C14/d_31_-C16 (Figure 6(a)) and between d_1_-C2/d_31_-C16 and d_23_-C12/d_31_-C16 (Figure 6(b)). Although VLCAD deficiency appeared to have a positive correlation between d_1_-C2/d_31_-C16 and d_23_-C12/d_27_-C14 (Figure 6(d)), this correlation was not statistically significant. There was no apparent correlation between d_1_-C2/d_31_-C16 and d_27_-C14/d_31_-C16 (Figure 6(c)).

## 4. Discussion

We demonstrated that the ratios of labeled acylcarnitines, as metabolites of loaded d_31_-hexadecanoic acid, were diagnostic for long-chain fatty acid oxidation defects and carnitine cycle disorders. Because it is not practical to check the viability of PBMCs collected from heparinized blood samples, we used the concentrations of d_31_-C16 and d_15_-octanoylcarnitine in the lysates without correction according to the cell count, in order to evaluate the quality of our test. Enzyme activities in PBMCs were occasionally lower than expected based on the disease severity. Furthermore, PBMCs are comprised of heterogenous cell subsets, including lymphocytes and monocytes, and the enzymatic functions of each subset have not been fully clarified. Therefore, we assumed that using ratios would allow us to overcome the influence of different components of PBMCs between infants and adults.

Our test was simple and practical since we used flow injection MS/MS, which is applied in acylcarnitine analysis for NBS. Janzen et al. recently reported a study in which peripheral whole blood was incubated with [16-^2^H_3_, 15-^2^H_2_] hexadecanoic acid, and long-chain fatty acid oxidation defects and carnitine cycle disorders were then evaluated using increased amounts of labeled acylcarnitines [6]. In their study, there were no significant increases in the concentrations of labeled C14 in patients with VLCAD deficiency compared with controls. Despite incubation with a labeled saturated fatty acid, they diagnosed VLCAD deficiency based on changes in the concentration of labeled C14:1, an unsaturated fatty acid. Moreover, it is difficult to evaluate *β*-oxidation capacity because nonlabeled acetylcarnitine was formed after incubation with [16-^2^H_3_, 15-^2^H_2_] hexadecanoic acid.

Enzyme activity is generally evaluated by tracing changes in the amounts of substrate and product in a metabolic pathway. Enzymes involved in the *β*-oxidation system metabolize multiple substrates with different affinities [17, 18]. In this context, analysis of multiple substrate-to-product ratios is rational for assessment of enzyme activities. Previous reports have demonstrated that the profiles of long-chain acyl-CoA and medium-chain acyl-CoA in mouse brains were similar to those of acylcarnitines in cells [19]. Therefore, we analyzed acylcarnitines instead of acyl-CoA as a measure of intracellular fatty acid metabolism.

In patients with CPT-II deficiency, the conversion of long-chain acylcarnitines to acyl-CoAs is impaired, and acylcarnitine profiles in mitochondria are characterized by accumulation of long-chain acylcarnitines. Indeed, in our test, d_31_-C16 increased and d_27_-C14 decreased in CPT-II-deficient cells, resulting in decreased ratios of d_27_-C14/d_31_-C16 compared with control cells. Because d_27_-C14-acylcarnitine is thought to be produced from d_27_-C14-acyl-CoA, which was formed through *β*-oxidation of d_31_-C16-acyl-CoA, the decrease in the ratio of d_27_-C14/d_31_-C16 could reflect the extent of enzyme activity impairment. Notably, the ratio of d_23_-C12/d_27_-C14 was also decreased in CPT-II-deficient cells, although the underlying mechanism remains unclear. The decreased d_27_-C14/d_31_-C16 ratio seemed to be a specific index for diagnosis of CPT-II deficiency. However, the ratio for patient no. 6 (Tables 1 and S1) who developed severe metabolic crisis overlapped with that for the controls. Based on the mutations in the CPT-II gene (p.S113L and p.C445R), the patient no. 6 was classified as having the mild myopathic form of CPT-II deficiency [13]. Although NBS data were not available for this case, it likely would be difficult to diagnose this patient in NBS, based on our recent experience with similar cases (data not shown). Of note, the d_23_-C12/d_31_-C16 ratio could distinguish this patient with CPT-II deficiency from controls. Patient no. 1 and patient no. 3 were both missed by the former screening marker (Tables 1 and S1), while diagnosis of these patients was possible using our proposed ratios. Therefore, it is necessary to comprehensively evaluate these two indices for the diagnosis of CPT-II deficiency.

In patients with VLCAD deficiency, *β*-oxidation of long-chain acyl-CoAs is impaired, and long-chain acylcarnitines, derived from long-chain acyl-CoAs, are then accumulated in mitochondria. Increased C14:1-acylcarnitine is diagnostic for VLCAD deficiency, and C14:1-acylcarnitine is thought to be derived from unsaturated long-chain fatty acids [20]. Because labeled oleic acid was not loaded on VLCAD-deficient cells, we could not detect an increase in d_27_-C14:1. However, accumulation of d_27_-C14 and reduction of d_23_-C12, which were measured as increased d_27_-C14/d_31_-C16 and decreased d_23_-C12/d_27_-C14 ratios, respectively, were observed in VLCAD-deficient cells. The combination of increased d_27_-C14/d_31_-C16 and decreased d_23_-C12/d_27_-C14 was required for diagnosis of VLCAD deficiency.

In patients with TFP deficiency, long-chain hydroxyacylcarnitines are accumulated in mitochondria due to impairment of three steps following dehydrogenation in *β*-oxidation. In our test, d_29_-C16-OH increased, and an increase in the d_29_-C16-OH/d_31_-C16 ratio was diagnostic.

In patients with CPT-I deficiency, synthesis of long-chain acylcarnitines from acyl-CoA and carnitine is impaired. Thus, free carnitine levels increased, and long-chain acylcarnitine levels decreased in mitochondria. In contrast, medium-chain fatty acids are transported into mitochondria independent of CPT-I and can be used to treat patients with CPT-I deficiency. In our test, values of d_7_-C4 and d_31_-C16 in PBMCs of patients with CPT-I deficiency decreased compared with those in controls after incubation with d_31_-hexadecanoic acid (Table S2). However, in controls, d_31_-C16 levels were distributed over a wide range and sometimes fell to low levels, possibly due to low cell counts or decreased cell viability. Therefore, to clearly distinguish CPT-I-deficient cells from control cells with low viability, we performed two different loading tests using only d_31_-hexadecanoic acid or both d_15_-octanoic acid and d_31_-hexadecanoic acid. After incubation with two labeled fatty acids, d_7_-C4 increased despite the low d_31_-C16 levels, and the d_7_-C4/d_31_-C16 ratio was significantly elevated in patients compared with controls. Furthermore, a comparison of the d_7_-C4/d_31_-C16 ratios between those tests clearly showed the differences between patients and controls and seemed to be useful for diagnosis of CPT-I deficiency.

Through the *β*-oxidation system, eight d_1_-C2s are theoretically produced from one d_31_-hexadecanoic acid. Although d_1_-C2 existed naturally at about 15% of the amount of d_0_-C2 in our experiment, increased d_1_-C2 was observed after the d_31_-hexadecanoic acid load. Therefore, the ratio of d_1_-C2 to d_31_-C16 in cells loaded with d_31_-hexadecanoic acid may indicate the capability of *β*-oxidation from long-chain acyl-CoA. Indeed, the d_1_-C2/d_31_-C16 ratios decreased in patients with CPT-II deficiency, VLCAD deficiency, and TFP deficiency compared with controls.

In CPT-II deficiency, d_1_-C2/d_31_-C16 was significantly correlated with d_27_-C14/d_31_-C16 and d_23_-C12/d_31_-C16. In VLCAD deficiency, d_1_-C2/d_31_-C16 seemed to be correlated with d_25_-C12/d_27_-C14, although the correlation was not statistically significant. Moreover, there were no correlations between d_1_-C2/d_31_-C16 and d_27_-C14/d_31_-C16. Because of the small number of patients in this study, relationships among diagnostic indices, *β*-oxidation indices, enzyme activities, and genotypes should be further investigated.

## 5. Conclusion

The method established in this study was found to be valuable for establishing rapid diagnosis of fatty acid oxidation disorders and carnitine cycle disorders, including CPT-I deficiency, in suspicious cases identified by NBS and high-risk patient screening. Thus, this method could complement enzyme activity measurement and gene analysis. Further studies are needed to verify the usefulness of the d_1_-C2/d_31_-C16 ratio as a *β*-oxidation ability for assessment of enzyme activity or disease severity.

## Figures and Tables

**Table 1:** Profiles of patients with VLCAD deficiency, CPT-II deficiency, and TFP deficiency.

Patients		Clinical form	Screening markers of acylcarnitine in DBS for NBS (*μ*M)					Diagnostic markers of acylcarnitine in serum (*μ*M)				Residual enzyme activity^a^ (%)
Diagnosis	No.		C14:1 <0.4^∗^	C16 <6.3^†^	C16-OH <0.05^∗^	C18:1-OH <0.05^∗^	C0/(C16+C18) <100^∗^	C14:1 0.08 (0.04)^‡^	C16 0.09 (0.04)^‡^	C16-OH 0.005 (0.001)^‡^	C18:1-OH 0.005 (0.001)^‡^	
VLCAD deficiency	1^b^	Severe neonatal	4.08					ND				0.017
	2	Myopathic	4.57					3.42				11.5
	3	Myopathic	2.72					6.49				5.6
	4	Myopathic	ND					2.62				9.4
	5	Myopathic	ND					1.19				NT

CPT-II deficiency	1	Infantile		3.45					3.01			NT
	2	Infantile		9.93					4.14			NT
	3	Myopathic		5.07					1.57			NT
	4^c^	Myopathic		12.20					3.02			NT
	5^d^	Myopathic		10.82					2.17			NT
	6^e^	Myopathic		ND					0.62			NT

TFP deficiency	1	Infantile			ND	ND				0.220	0.180	NT
	2^f^	Myopathic			ND	ND				0.059	0.076	NT
	3^g^	Myopathic			ND	ND				0.093	0.129	NT

CPT-I deficiency	1	Asymptomatic					267					NT
	2	Infantile					133					NT
	3	Asymptomatic					127					NT

NT: not tested; ND: no data; C0: free carnitine; C14:1: tetradecenoylcarnitine; C16: hexadecanoylcarnitine; C16-OH: hydroxy-hexadecanoylcarnitine; C18:1-OH: hydroxy-octadecenoylcarnitine; C18: octadecanoylcarnitine. ^∗^Cutoff value in NBS. ^†^Cutoff value in NBS when these patients were diagnosed. ^‡^Mean (SD) of 35 controls without starvation. ^a^Enzyme activity is presented as a percentage of the mean of the normal control [7]. ^b^Patient details were previously reported by Yamamoto et al. [14]. ^c,d^Nos. 4 and 5 with CPT-II deficiency were siblings. ^e^Patient details were previously reported by Matsumoto et al. [13]. ^f,g^Nos. 2 and 3 with TFP deficiency were siblings.

## Data Availability

The data used to support the findings of this study are available from the corresponding author upon request.

## Supplementary Materials

Supplementary Materials Table S1: acylcarnitine analysis of the fatty acid oxidation test of patients with VLCAD deficiency, CPT-II deficiency, and TFP deficiency. In most patients with VLCAD deficiency, CPT-II deficiency, and TFP deficiency, d_27_-C14, d_31_-C16, and d_29_-C16-OH were increased, respectively. The ratios in Figures 2, 3, 5, and 6 were calculated using the data listed in this table. Table S2: acylcarnitine analysis of the fatty acid oxidation test of patients with CPT-I deficiency. In patients with CPT-I deficiency, d_7_-C4 levels after loading both d_31_-hexadecanoic acid and d_15_-octanoic acid were higher than that after loading d_31_-hexadecanoic acid alone. The ratios in Figure 4 were calculated using the data listed in this table.
