# Supplementary material for: Evaluation of Metabolic Defects in Fatty Acid Oxidation Using Peripheral Blood Mononuclear Cells Loaded with Deuterium-Labeled Fatty Acids
Source: Dis Markers. 2019 Feb 7;2019:2984747. doi: 10.1155/2019/2984747 (PMC6383405; doi:10.1155/2019/2984747)
Supplement: Supplementary Materials — Table S1: acylcarnitine analysis of the fatty acid oxidation test of patients with VLCAD deficiency, CPT-II deficiency, and TFP deficiency. In most patients with VLCAD deficiency, CPT-II deficiency, and TFP deficiency, d27-C14, d31-C16, and d29-C16-OH were increased, respectively. The ratios in Figures 2, 3, 5, and 6 were calculated using the data listed in this table. Table S2: acylcarnitine analysis of the fatty acid oxidation test of patients with CPT-I deficiency. In patients with CPT-I deficiency, d7-C4 levels after loading both d31-hexadecanoic acid and d15-octanoic acid were higher than that after loading d31-hexadecanoic acid alone. The ratios in Figure 4 were calculated using the data listed in this table. [file 2984747.f1.pdf]

Supplementary Materials

Table S1. Acylcarnitine analysis of fatty acid oxidation test of patients with VLCAD deficiency, CPT-II deficiency and TFP deficiency

Patients			Acylcarnitine analysis of fatty acid oxidation test (nmol/ml lysate)				
Diagnosis	No.	Age at the examination of fatty acid oxidation test	d_1-C2 2.98 (0.93)*	d_{23}-C12 0.15 (0.07)*	d_{27}-C14 0.22 (0.10)*	d_{31}-C16 0.78 (0.33)*	d_{29}-C16-OH 0.01 (0.01)*
VLCAD deficiency	1	0	1.16	0.15	5.75	4.85	0.02
	2	3	0.41	0.11	2.80	4.15	0.02
	3	11	0.21	0.02	0.39	0.51	0.01
	4	19	1.25	0.15	1.20	1.74	0.01
	5	32	0.49	0.06	0.69	0.89	0.01
CPT-II deficiency	1	0	2.79	0.05	0.22	20.51	0.02
	2	1	0.38	0.00	0.03	2.47	0.01
	3	1	2.54	0.04	0.12	9.73	0.01
	4	2	1.62	0.03	0.21	21.76	0.01
	5	4	0.09	0.00	0.01	1.08	0.00
	6	9	0.18	0.01	0.04	0.24	0.01
TFP deficiency	1	17	1.46	0.22	0.95	2.27	0.33
	2	20	2.11	0.34	1.25	2.55	0.62
	3	25	3.27	0.82	3.39	7.90	2.52

*Mean (SD) of 23 healthy controls.

Table S2. Acylcarnitine analysis of fatty acid oxidation test of patients with CPT-I deficiency

Patients with CPT-I deficiency	Age at the examination of fatty acid oxidation test	Acylcarnitine analysis of fatty acid oxidation test						
		loaded with d_{31}-hexadecanoic acid (nmol/ml lysate)			loaded with d_{31}-hexadecanoic acid and d_{15}-octanoic acid (nmol/ml lysate)			
No.		d_1-C2 2.98 (0.93)*	d_7-C4 0.39 (0.20)*	d_{31}-C16 0.78 (0.33)*	d_1-C2 3.18 (0.95)†	d_7-C4 0.48 (0.22)†	d_{15}-C8 0.05 (0.03)†	d_{31}-C16 0.49 (0.17)†
1	0	0.74	0.40	0.24	0.98	1.42	0.01	0.14
2	5	0.18	0.03	0.01	0.14	0.15	0.35	0.01
3	5	0.99	0.03	0.08	1.11	0.30	0.01	0.06

*Mean (SD) of 23 healthy controls.

†Mean (SD) of 8 other healthy controls.
